# Helios, CD73 and CD39 Induction in Regulatory T Cells Exposed to Adipose Derived Mesenchymal Stem Cells

**DOI:** 10.22074/cellj.2020.6313

**Published:** 2019-10-14

**Authors:** Maryam Fakhimi, Abdol-Rasoul Talei, Abbas Ghaderi, Mojtaba Habibagahi, Mahboobeh Razmkhah

**Affiliations:** 1. Shiraz Institute for Cancer Research, School of Medicine, Shiraz University of Medical Sciences, Shiraz, Iran; 2. Department of Immunology, School of Medicine, Shiraz University of Medical Sciences, Shiraz, Iran; 3. Breast Diseases Research Center (BDRC), Shiraz University of Medical Sciences, Shiraz, Iran

**Keywords:** Adipose-Derived Mesenchymal Stem Cell, Breast Cancer, Immunomodulatory Effects, Regulatory T Cells

## Abstract

**Objective:**

Mesenchymal stem cells (MSCs) have prominent immunomodulatory roles in the tumor microenvironment. The current study intended to elucidate Treg subsets and their cytokines after exposing naïve T lymphocytes to adipose- derived MSCs (ASCs).

**Materials and Methods:**

In this experimental study, to obtain ASCs, breast adipose tissues of a breast cancer patient and a normal individual were used. Magnetic cell sorting (MACS) was employed for purifying naïve CD4^+^T cells from peripheral blood of five healthy donors. Naïve CD4^+^T cells were then co-cultured with ASCs for five days. The phenotype of regulatory T cells (Tregs) and production of interleukine-10 (IL-10), transforming growth factor beta (TGF-β) and IL-17 were assessed using flow cytometry and ELISPOT assays, respectively.

**Results:**

CD4^+^CD25^-^FOXP3^+^CD45RA^+^Tregs were expanded in the presence of cancer ASCs but CD4^+^CD25^+^Foxp3^+^CD45RA^+^regulatory T cells were up-regulated in the presence of both cancer- and normal-ASCs. This up-regulation was statistically significant in breast cancer-ASCs compared to the cells cultured without ASCs (P=0.002). CD4^+^CD25^+^ FOXP3^+^Helios^+^, CD4^+^CD25^-^FOXP3^+^Helios^+^and CD25^+^FOXP3^+^CD73^+^CD39^+^Tregs were expanded after co-culturing of T cells with both cancer-ASCs and normal-ASCs, while they were statistically significant only in the presence of cancer-ASCs (P<0.05). Production of IL-10, IL-17 and TGF-β by T cells was increased in the presence of either normal- or cancer-ASCs; however, significant effect was only observed in the IL-10 and TGF-β of cancer-ASCs (P<0.05).

**Conclusion:**

The results further confirm the immunosuppressive impacts of ASCs on T lymphocytes and direct them to specific regulatory phenotypes which may support immune evasion and tumor growth.

## Introduction

Mesenchymal stem cells (MSCs) are multipotent adult stem cells and their primitive origin is mesoderm. They possess self-renewal ability and are capable of differentiating into not only mesodermal cell lineage, but also ectodermic and endodermic cell lineages (1, 2). MSCs have the ability to home and engraft at sites of injury in the pathological conditions such as inflammation, neoplasia and tissue repair. These cells are recruited into tumor microenvironment in response to cytokines and chemokines produced by tumor cells. Within the tumor mass, MSCs can differentiate into fibroblasts, myofibroblasts, pericytes and carcinomaassociated fibroblast and cooperate with endothelial and inflammatory cells exacerbating tumor status (3, 4).

The anti-inflammatory and immune-modulating properties of MSCs have been shown in many studies. For instance, prohibiting effector T-cells activation along with increase in regulatory T cells (Tregs) population (5, 6) are known as important immune-modulating mechanisms. The suppression mechanism of T-cells by MSCs could be direct (cell-cell contact) or may occur indirectly by secreting soluble factors such as nitric oxide (NO), indoleamine 2,3-dioxygenase (IDO), prostaglandin E2 (PGE2), interleukine-10 (IL-10) and transforming growth factor beta (TGF-β) (7, 8). They inhibit activation and function of T-cells by down-regulating major histocompatibility complex (MHC) class II molecules and IL-12, decreasing interferon-γ (IFN-γ) and inducing generation of Tregs through IL-10 production, besides of releasing human leukocyte antigen-G (HLA-G) (9, 10). Tregs are generally characterized by the expression of forkhead box transcription factor (FOXP3) and play prominent roles in peripheral tolerance and controlling immune responses, towards the autoimmune diseases, allergies, infection-induced organ pathology and tumors (11, 12).

Soluble mediators such as TGF-β, IL-10 and IL-35 in addition to immunosuppressive metabolites, including adenosine production, are responsible for the suppressive mechanisms of Tregs (13-15). Naturally occurring Treg cells and antigen-induced CD4^+^CD25^+^FOXP3^+^ Tregs have been widely studied. However, other additional subsets and markers of Treg cells have received less attention in identification and characterization of these cells (16). CD45RA, CD25, CD73, CD39 and Helios are among the most important markers of Treg cells, whose roles have recently been identified (17). CD39 and CD73 are recognized as markers of Tregs with the capability of circular adenosine monophosphate (cAMP) or adenosine mediated suppression (18). Recent studies confirmed that MSC-exposed Tregs carry more immunosuppressive properties than Tregs co-cultured without MSCs (19). Here, we further clarified Treg subsets through assessment of CD45RA, CD73, CD39 and Helios expression after adipose derived mesenchymal stem cell (ASC)-T cell crosstalk. Then, expression level of IL-10, TGF-β and IL-17 were determined in T cells co-cultured in the presence of ASCs.

## Materials and Methods

This experimental study was approved by the Ethics Committee of Shiraz University of Medical Sciences (Shiraz, Iran, Code No. 9113). All donors were provided written and signed informed consent to take part in this study.

### Isolation, culture and characterization of human adipose-derived mesenchymal stem cells

ASCs were provided from breast adipose tissues of one breast cancer patient and one normal individual undergoing mammoplasty surgery as previously explained (15, 20, 21). Briefly, fragments of adipose tissue were washed with phosphate buffered saline (PBS), minced and digested using 0.2% collagenase type I (Gibco, USA) at 37˚C for about 45 minutes. The digested materials were centrifuged at 400 g for 10 minutes. The stromal vascular fraction (SVF) was separated using Ficoll gradient (Biosera, UK) through centrifugation at 400 g for 20 minutes. The cells were washed with PBS and plated into T-25 plastic cell culture flasks in Dulbecco’s Modified Eagle’s Medium (DMEM, Biosera, UK) containing 10% fetal bovine serum (FBS, Gibco, USA) and 1% penicillin/ streptomycin (Gibco, USA). The cultured cells were incubated at 37˚C and 5% CO_2_ with 95% humidity. Following 24 hours incubation, non-adherent cells were discarded. The medium was exchanged every 72 hours. The harvested cells were sub-cultured and used for further experiments. To characterize the cells with flow-cytometer, ASCs were stained with fluorescein isothiocyanate (FITC)-conjugated mouse anti-human CD45, CD34 and CD14 (BD Biosciences, USA) as well as phycoerythrin (PE)-conjugated mouse antihuman CD90, CD105, CD44, CD73 (BD Biosciences). To evaluate differentiation capacity of the cells, they were treated with conditioned medium for osteocyte, chondrocyte and adipocyte differentiation, as previously described (15, 20, 21). ASCs isolated from the breast cancer patient and normal subject are hereafter called cancer-ASCs and normal-ASCs in this article.

### Isolation of naïve CD4^+^ T cells from human peripheral blood mononuclear cells

Peripheral blood mononuclear cells (PBMCs) were isolated by density gradient centrifugation using Ficoll gradient (Biosera, USA) from six healthy donors with respectively the mean ± standard deviation (SD) and median age of 33.8 ± 2.7 and 35 years old. To exclude monocytes from mononuclear cells, isolated PBMCs were cultured at 37˚C for 45 minutes. After incubation time, the remaining flout cells were collected as peripheral blood lymphocytes (PBLs). Separation of naïve CD4^+^ T cells was performed using the Human Naive CD4^+^ T cell Isolation Kit II (Miltenyi Biotec, Germany). In brief, highly pure naïve CD4^+^ T cells were achieved by depletion of magnetically labeled non-CD4^+^ T cells and CD45RO^+^ memory T cells using a cocktail of biotin-conjugated antibodies against CD8, CD14, CD15, CD16, CD19, CD25, CD34, CD36, CD45RO, CD56, CD123, T cell receptor (TCR) γ/γ, HLA-DR, CD235a and anti-Biotin micro-beads (Miltenyi Biotec, Germany). The cell subset purity was regularly tested using flow cytometer, for the expression of CD4 and CD45RA.

### Co-culture of adipose-derived mesenchymal stem cells and naïve CD4^+^ T cells

Naïve CD4^+^ T cells (25×10^4^) were directly cultured with ASCs (25×10^3^) at a ratio of 10 to 1 in RPMI 1640 (Biosera, UK) containing 10% FBS, 1% penicillin/streptomycin, 20 ng/ml phytohemagglutinin (PHA, Roche, Germany) and 7% autologous serum of healthy donors. The culture was incubated at 37˚C and 5% CO_2_ with 95% humidity for five days.

### Flow cytometry analysis

T cells were removed from culture and subjected to flow cytometer for characterization. To blockade Fc receptors, all cells were incubated for 10 minutes at 4˚C with 10 μl/ml human serum before staining with fluorescent antibodies. Fluorescent antibodies and the respective isotype controls were then added. Flow cytometry analysis was performed with a FACSCalibur (BD Biosciences) using directly labeled monoclonal Abs (mAbs), PerCP mouse anti-human CD4, FITC mouse anti-human CD25, APC mouse anti-human CD45RA, PerCP-CYTM5.5 mouse anti-human CD73, APC mouse anti-human CD39, PE mouse anti-human FOXP3 (BD Biosciences), APC antimouse/ human Helios (Biolegend, Germany) and isotype control antibodies, all according to the manufacturers’ protocol. The collected data were analyzed using FlowJo 7.6 software.

### Measurement of cytokine production by enzyme linked immunosorbent spot assay

ASCs (25×10^3^) were directly co-cultured with isolated naïve CD4^+^ T cells (25×10^4^) in RPMI 1640 containing 10% FBS, 1% penicillin/streptomycin, 20 ng/ml PHA and 7% autologous serum of healthy donors. After five days, T cells were extracted from the culture and the production of cytokines including IL-10 (U-CyTech Biosciences, Netherland), TGF-β (R&D, USA) and IL-17 (U-CyTech Biosciences, Netherland) was measured. The ELISPOT assays were performed according to the manufacturers’ instruction.

### Statistical analysis

The data are represented as mean ± standard error mean (SEM). All statistical analyses were performed using statistical package for the social sciences (SPSS, Chicago, IL, USA) software version 16.0, nonparametric Mann-Whitney U test, Friedman and Dunn’s and Kruskal-Wallis H tests. All graphs were plotted and evaluated by means of FlowJo 7.6.2 and GraphPad Prism 5 software. The P<0.05 were considered statistically significant.

**Fig 1:**
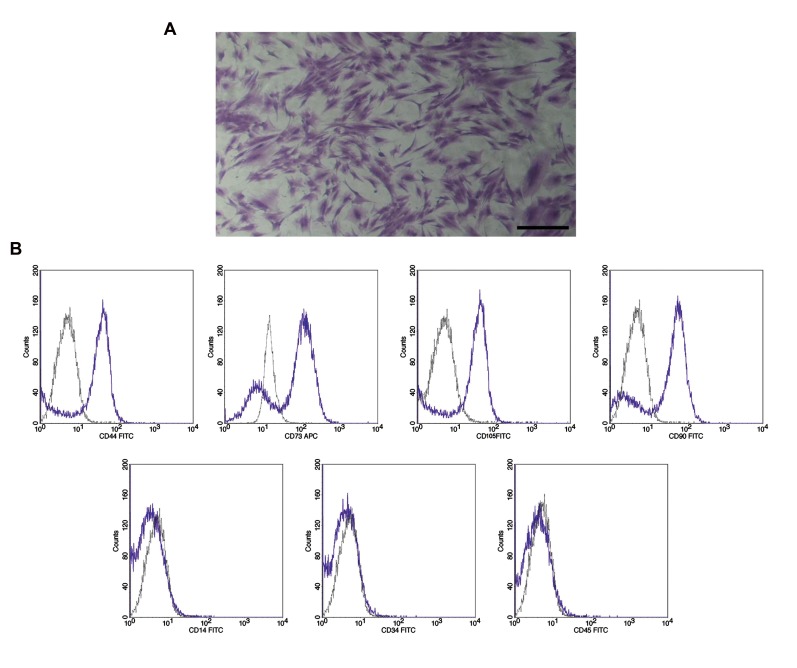
Morphological characterization of adipose-derived mesenchymal stem cells (ASCs) as well as flow cytometry analysis. **A.** ASCs were appeared with spindle shape in culture (scale bar: 100 μm) and **B.** The flow cytometry analysis of ASCs illustrate that more than 90% of all cancer and normal ASCs were positive for stem cell specific markers including CD44, CD105, CD73 and CD90, but they were negative for the expression of hematopoietic specific markers such as CD14, CD45 and CD34.

## Results

### Adipose-derived mesenchymal stem cells isolation and characterization

ASCs had adhesion properties to the bottom of culture flasks and they were appeared as a homogenously spindle shaped population (Fig .1A). The flow cytometry analysis of ASCs revealed that more than 90% of all cancer and normal ASCs were positive for the stem cell specific markers, including CD90, CD105, CD44 and CD73, but they were negative for the expression of hematopoietic specific markers, such as CD45, CD34 and CD14 (Fig .1B).

### Purity of the isolated naïve CD4^+^ T cells

**Fig 2:**
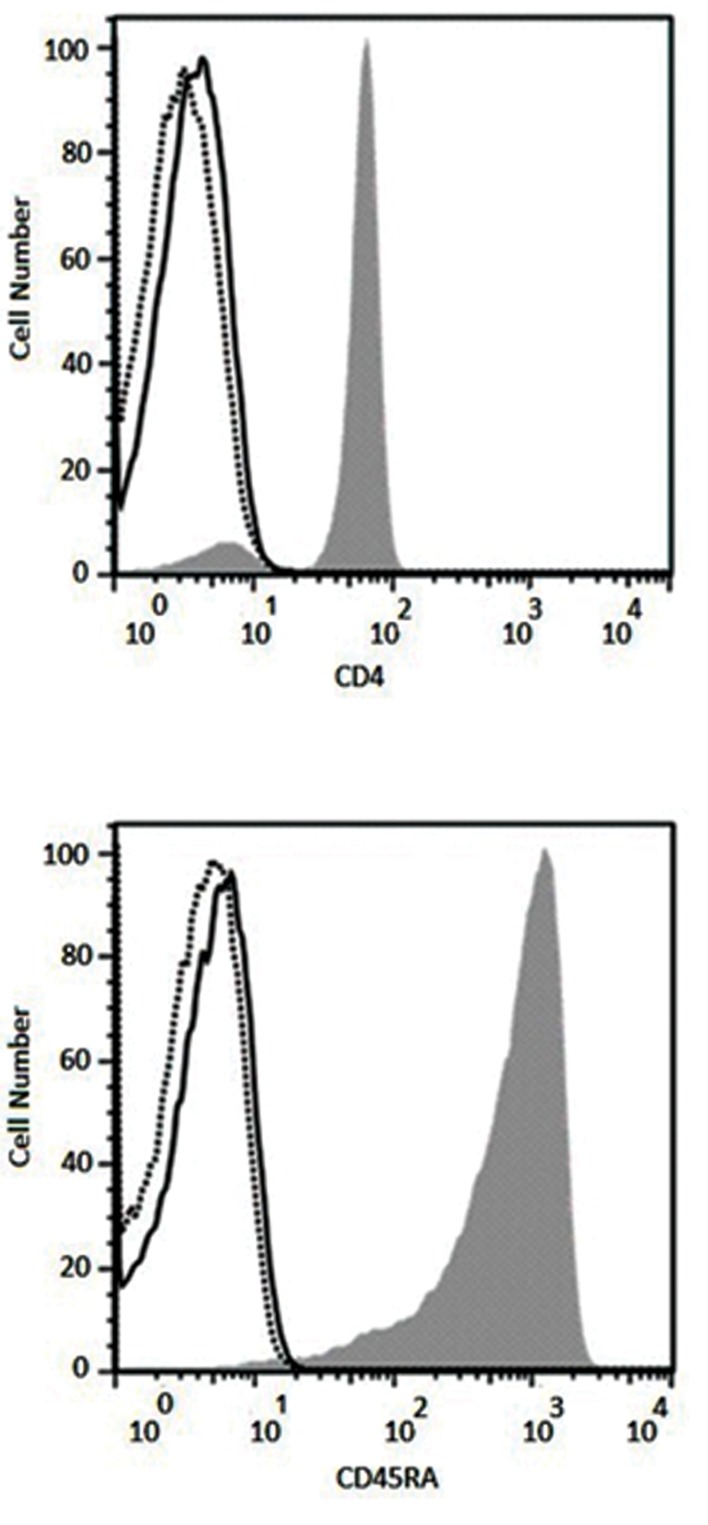
Purity of the isolated naïve CD4^+^ T cells was evaluated using flow cytometry for the expression of CD4 and CD45RA (solid histograms). Results illustrate more than 96% purity for CD4^+^ and CD45RA^+^ cells. Solid and lined histograms represent isotype control and unstained cells, respectively.

After isolating naïve CD4^+^ T cells from peripheral blood of healthy donors, their purity were verified by CD4 and CD45RA expression analyses using flow cytometry. Results showed more than 96% purity for CD4^+^ CD45RA^+^ cells (Fig .2).

### Phenotypic analysis of harvested regulatory T cells

Phenotype of the harvested Tregs was studied more in detail, as CD45RA^+^ and Helios^+^ T cells were determined in the population of CD4^+^CD25^+^FOXP3^+^ and CD4^+^CD25^-^ FOXP3^+^ T lymphocytes. CD73^+^CD39^+^ T lymphocytes were also assessed in the population of CD25^+^ FOXP3^+^ and CD25^-^ FOXP3^+^ cells.

### CD45RA^+^ T cells in the population of CD4^+^CD25^+^ FOXP3^+^ and CD4^+^CD25^-^FOXP3^+^ cells

**Fig 3:**
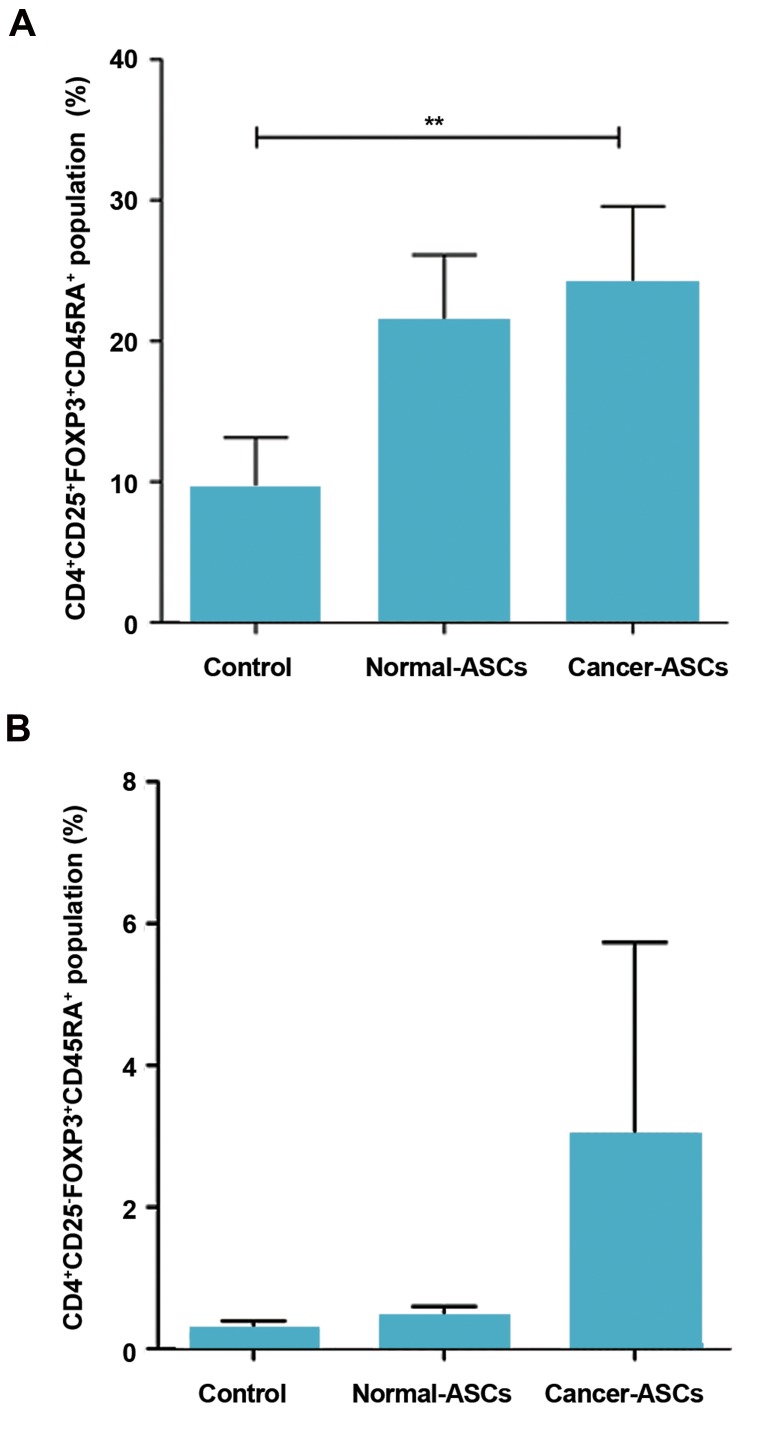
The percentage of CD45RA^+^CD4^+^CD25^+^FOXP3^+^ and CD45RA^+^CD4^+^CD25^-^ FOXP3^+^ Tregs population. After five days culturing of naïve CD4^+^ T cells with adipose-derived mesenchymal stem cells (ASCs), the percentage of A. CD45RA^+^CD4^+^CD25^+^FOXP3^+^ T cells and B. CD45RA^+^CD4^+^CD25^-^Foxp3^+^ T cells was evaluated by flow cytometry method. The results illustrate mean ± SEM of cell percentages. **; P<0.01 compared to the control group.

After five days co-culturing naïve T cells with normal- and cancer-ASCs, CD45RA^+^ T cells were investigated in the population of CD4^+^CD25^+^FOXP3^+^ and CD4^+^CD25^-^FOXP3^+^ cells. Results showed that the effect of cancer-ASCs was more significant than normal- ASCs on augmenting CD45RA^+^ cells, compared to the control group (P=0.002). Mean ± SEM percentage of CD4^+^CD25^+^FOXP3^+^CD45RA^+^ phenotype was 24.05 ± 5.5%, 21.36 ± 4.7%, and 9.51 ± 3.6% respectively after co-culturing with cancer-ASCs and normal-ASCs and in the control group (Fig .3A). Although naïve T cells coculture with cancer-ASCs resulted in approximately twofold expansion of CD45RA^+^ T cells in the population of CD4^+^CD25^-^FOXP3^+^ cells compared to the control group, this expansion was not statistically significant (P>0.05, Fig .3B).

### Helios^+^ T cells in the population of CD4^+^CD25^+^FOXP3^+^ and CD4^+^CD25^-^FOXP3^+^ cells

**Fig 4:**
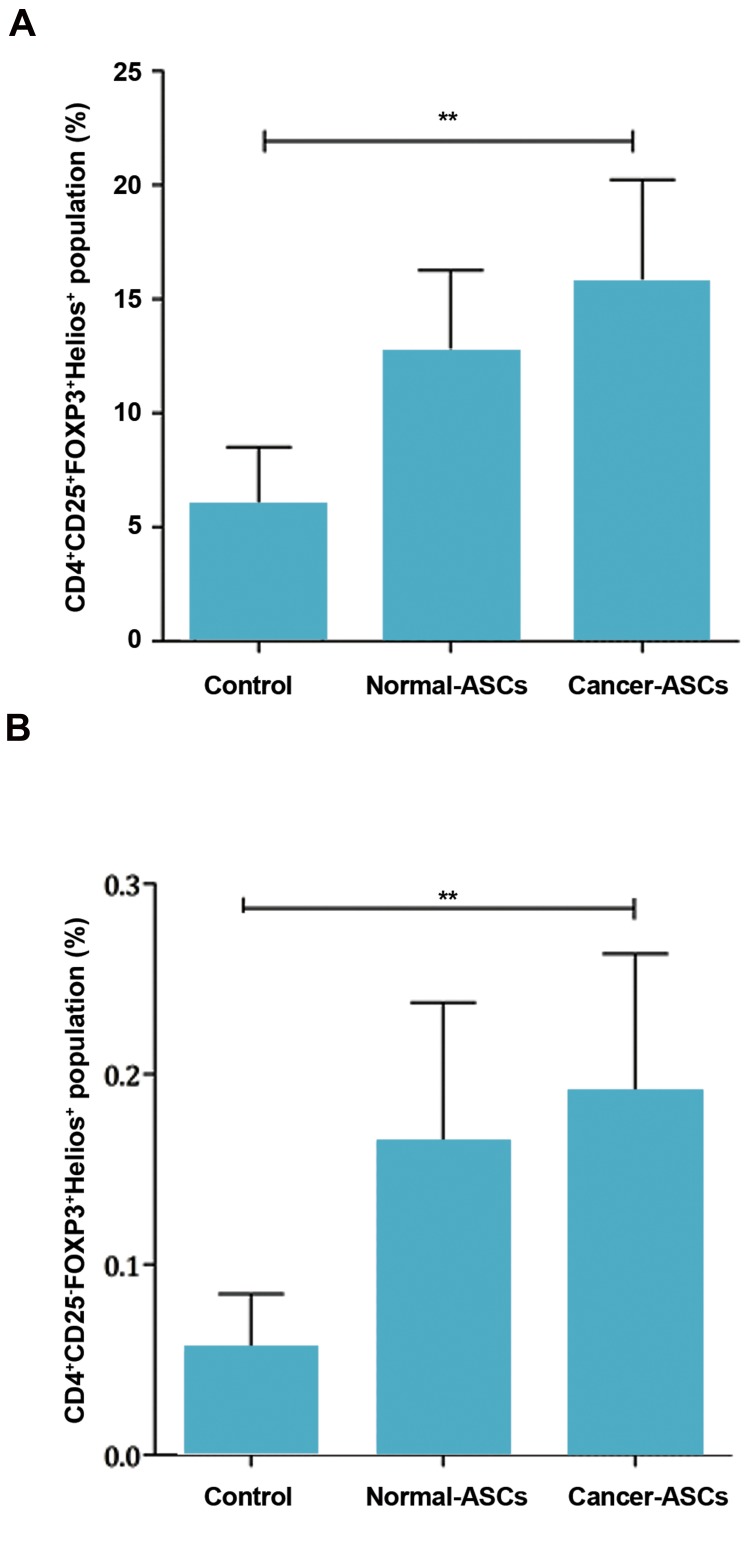
Percentage of the Helios^+^ Tregs population. After five days culturing of naïve CD4^+^ T cells with adipose-derived mesenchymal stem cells (ASCs). Percentage of A. The CD4^+^CD25^+^FOXP3^+^Helios^+^ T cells was evaluated by flow cytometry method and B. Percentage of the CD4^+^CD25^-^-FOXP3^+^Helios^+^ T cells population was also determined. The results illustrate mean ± SEM of cell percentages. **; P<0.01 compared to the control group.

Presence of normal-ASCs and cancer-ASCs in the culture increased Helios^+^ T cells in the population of CD4^+^CD25^+^FOXP3^+^ cells. The results showed that effect of cancer-ASCs was more significant than normal-ASCs on augmenting Helios^+^ cells compared to the control group (P=0.005). Mean ± SEM percentage of T cells with CD4^+^CD25^+^FOXP3^+^Helios^+^ phenotype were 15.59 ± 4.6%, 12.63 ± 3.6%, and 5.90 ± 2.6% in the presence of cancer-ASCs and normal-ASCs and in the control group, respectively. (Fig .4A).

Helios^+^ T cells, in the population of CD4^+^CD25^-^ FOXP3^+^, were increased in the presence of normal- ASCs and cancer-ASCs. Additionally, the effect of cancer-ASCs on augmentation of Helios^+^ T cells was significant, compared to the control group (P=0.005, Fig .4B).

CD4^+^CD25^+^FOXP3^-^Helios^+^ and CD4^+^CD25^+^FOXP3^+^Helios^-^ T lymphocytes were also investigated in this study. The results showed that co-culturing naïve CD4^+^ T cells with cancer- ASCs and normal-ASCs caused decreased population of CD4^+^CD25^+^FOXP3^-^Helios^+^ in comparison with the control group (P=0.028 and 0.028, respectively). Mean ± SEM percentage of CD4^+^CD25^+^FOXP3-Helios^+^ phenotype was 11.15 ± 0.6%, 12.47 ± 0.9% and 31.02 ± 4.2% after co-culturing with cancer-ASCs, normal-ASCs and control group, respectively. Changes in the phenotype of CD4^+^CD25^+^FOXP3^+^Helios^-^ T lymphocytes were not statistically significant (P>0.05).

#### CD73^+^CD39^+^ T cells in the population of CD25^+^FOXP3^+^ and CD25^-^FOXP3^+^ cells

**Fig 5:**
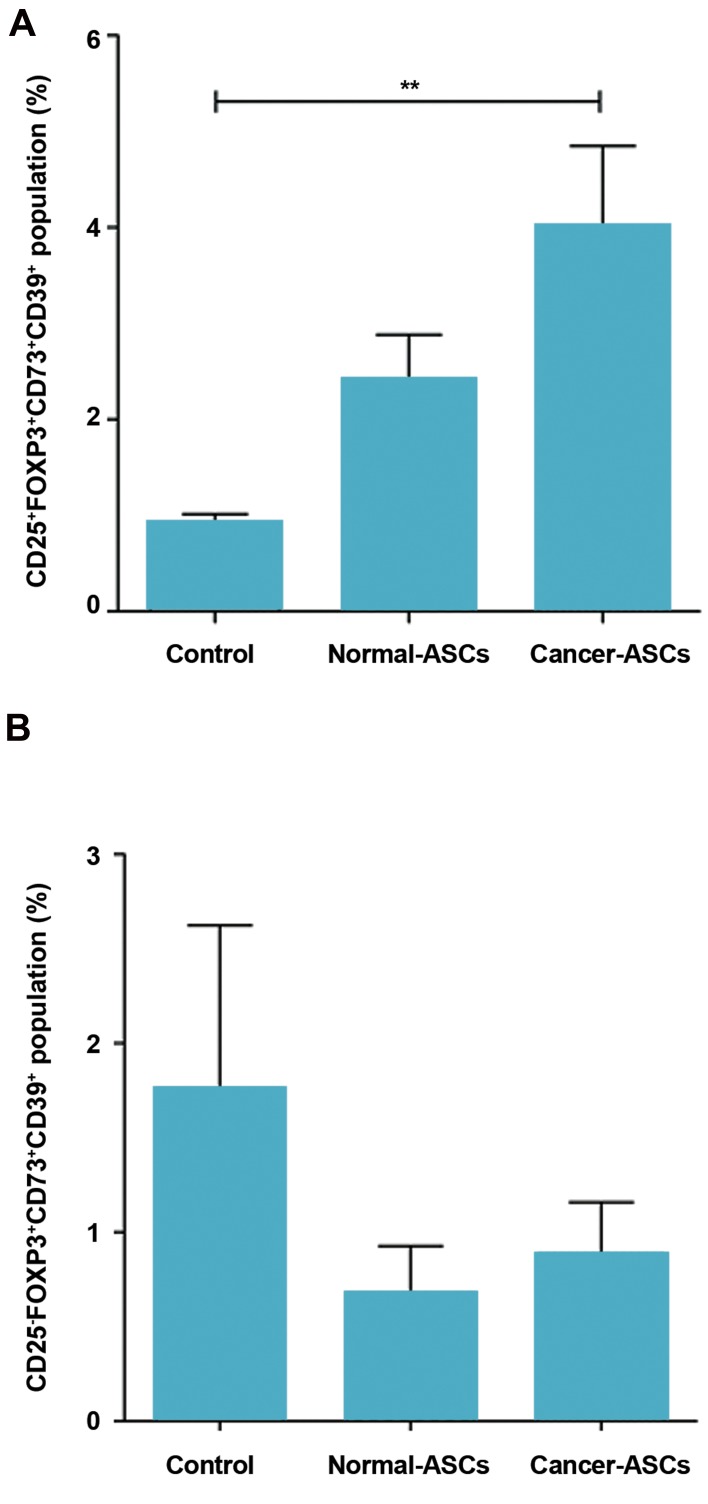
Percentage of the CD39^+^CD73^+^ in CD25^+^FOXP3^+^ and CD25^-^FOXP3^+^ T cells population subsequent to co-culturing with adipose-derived mesenchymal stem cells (ASCs). After five days culturing of naïve CD4^+^ T cells with ASCs, percentage of CD39^+^CD73^+^ T cells in A. CD25^+^FOXP3^+^ population and B. CD25^-^FOXP3^+^ population was evaluated by flow cytometry method. The results illustrate mean ± SEM. **; P<0.01 compared to the control group.

When we compared presence of the normal- ASCs and cancer-ASCs to the control group, the percentage of CD25^+^FOXP3^+^CD73^+^CD39^+^ phenotype was increased in both normal and cancer-ASCs. Although, this was only significant in the presence of cancer-ASCs (P=0.005). Mean ± SEM percentage of CD73^+^CD39^+^CD25^+^FOXP3^+^ T cells cultured with cancer-ASCs, normal-ASCs and control group were respectively 4.00 ± 0.84, 2.41 ± 0.47, and 0.93 ± 0.08. Phenotypic changes of CD39^+^CD73^+^ T cells in the population of CD2^-^-FOXP3^+^ was not significant (P> 0.05, Fig .5A, B).

The populations of CD39^+^CD73^+^CD25^+^FOXP3^+^ and CD39^+^CD73^+^CD25^-^FOXP3^+^ T lymphocytes were phenotypically compared when naïve CD4^+^ T cells cultured with cancer-ASCs, normal-ASCs and control group. Results showed significant phenotypic changes between these two populations when naïve CD4^+^ T cells were cultured with normal-ASCs and cancer- ASCs (P=0.017 and 0.008, respectively).

Mean ± SEM percentage of CD73^+^CD39^+^CD25^+^FOXP3^+^ and CD73^+^CD39^+^CD2^-^-FOXP3^+^ T cells, cultured with cancer-ASCs, were 4 ± 0.8 and 0.88 ± 0.2, respectively. Mean ± SEM percentage of CD73^+^CD39^+^CD25^+^FOXP3^+^ and CD73^+^CD39^+^CD25^-^FOXP3^+^ T cells, cultured with normal-ASCs, were respectively 2.4 ± 0.4 and 0.67 ± 0.2. Phenotypic changes of these two populations, when naïve CD4^+^ T cells cultured as control group, was not statistically significant (P >0.05).

#### Measurement of cytokine production by ELISPOT assay

**Fig 6:**
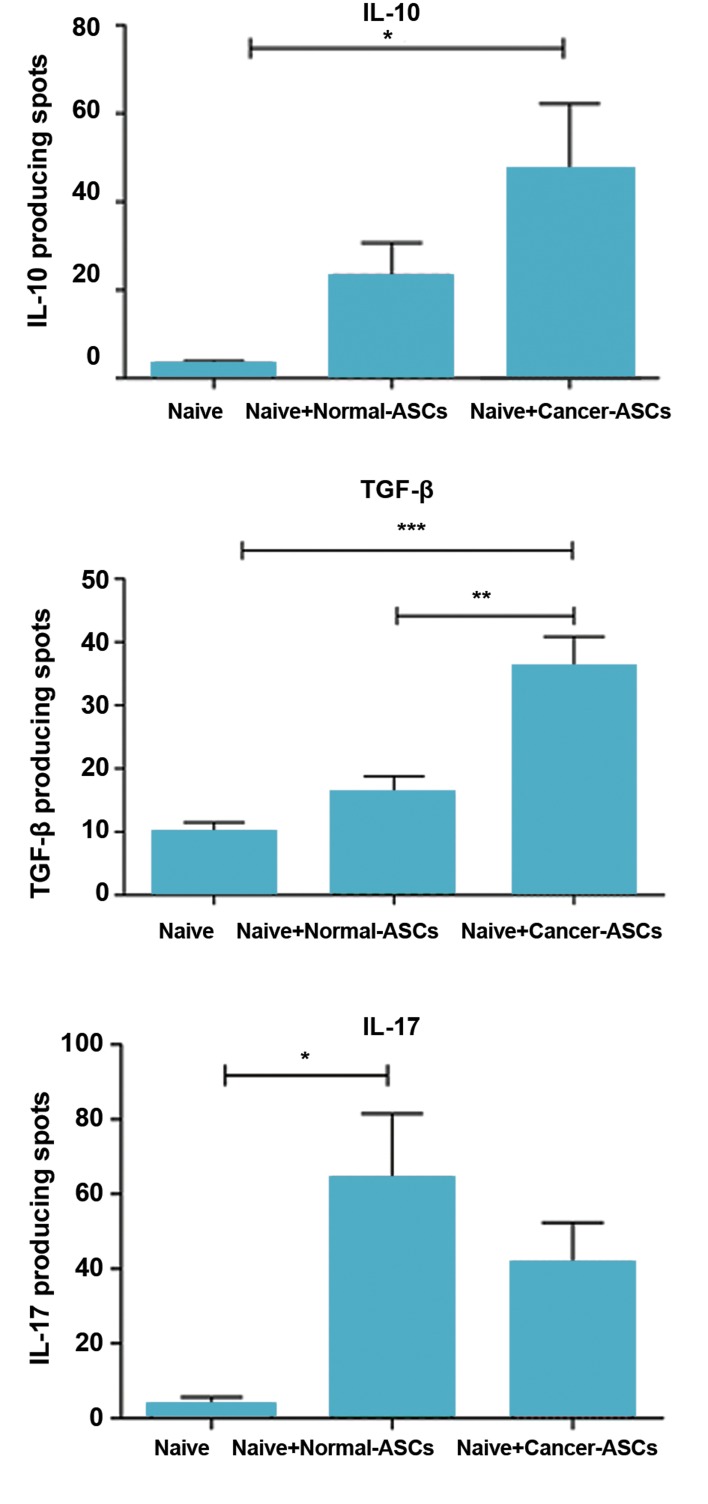
Producing interleukine-10 (IL-10), transforming growth factor beta (TGF-β) and IL-17 by T cell after co-culturing with adipose-derived mesenchymal stem cells (ASCs). After five days culturing of naïve CD4^+^ T cells with ASCs, IL-10, TGF-β and IL-17 produced by T cells were evaluated using ELISPOT method. The results illustrate mean ± SEM. *; P<0.05, **; P<0.01 and ***; P<0.001 compared to naïve T cells which were not cocultured with ASCs (control group).

Production of IL-10, TGF-β and IL-17 by T cells was evaluated in the presence and absence of ASCs, employing ELISPOT technique. The results showed that production of the cytokines was increased in the presence of ASCs. IL-10 production was increased by T cells, after exposure of naïve CD4^+^ T cells to either normal- or cancer-ASCs. This effect was statistically significant for the cancer-ASCs compared to the control group (P=0.038). Mean ± SEM number of IL-10 spots by T cells cultured with cancer-ASCs, normal- ASCs and control group were 47.17 ± 15.1, 23.17 ± 7.5, and 3.167 ± 0.72, respectively. TGF-β production of the T cells was increased in the presence of cancer-ASCs compared to normal-ASCs and absence of the ASCs (P=0.0006 and 0.003, respectively). Mean ± SEM number of TGF-β spots by T cells, cultured with cancer-ASCs, normal-ASCs and control group were respectively 36.08 ± 4.7, 15.96 ± 2.8, and 10 ± 1.4. IL-17 production of T cells was increased while they were co-cultured with ASCs and the effect of normal-ASCs on IL-17 production was significant compared to the naïve T cells which were not cultured with ASCs (P=0.015). Mean ± SEM of IL-17 spots by T cells, cultured with cancer-ASCs, normal-ASCs and control group were respectively 41.5 ± 1.8, 64.33 ± 17.2, and 3.7 ± 1.9 (Fig .6).

### Discussion

Previous studies have indicated that high infiltration of tumors by regulatory CD4^+^FOXP3^+^ T cells associate with poor prognosis in different types of solid tumors, through limiting antitumor immune responses (22). Low numbers of tumor infiltrating FOXP3^+^ T cells and high numbers of infiltrating CD8^+^ lymphocytes have been suggested as favorable prognostic markers for invasive ductal carcinoma of the breast (23). It is proposed that tumor promoting effects of Tregs is mostly mediated through their recruitment to the tumor microenvironment or local expansion rather than increased suppressive properties in the tumor sites (5, 22).

Among several cell types in the vicinity of tumor cells, MSCs are known as important players with immunomodulatory effects on both innate and adaptive immunity through direct cell to cell contact or secretion of soluble factors (5). Herein, ASC-naïve CD4^+^ T cell crosstalk was assessed and Treg subsets were subsequently further clarified in detail. The initial results disclosed that normal and cancer ASCs induced both CD25^+^ and CD25- Tregs, but cancer ASCs showed stronger effect for inducing CD25- phenotype compared to the normal ASCs. The observed expansion of CD25^-^-FOXP3^+^ Tregs in this study has been reported in previous researches (15, 21). CD25- T cells are a subset of Tregs induced by tumor and involved in tumor-induced immunosuppression (24). Yang et al. (25) realized that a specific proportion of intratumoral CD4^+^ T cells in non-Hodgkin lymphoma patients was CD4^+^CD25^-^FOXP3^+^ Tregs with the ability of suppressing T cells proliferation. In another study, both conventional and CD4^+^CD25^-^FOXP3^+^ Tregs were detected in tumor draining lymph nodes of colorectal cancer patients but CD25- T cells were characterized with lower suppressive properties (26). Thus according to the results of this part of our study, ASCs from breast cancer tissues may suppress immune responses through increasing population of CD4^+^CD25^-^FOXP3^+^CD45RA^+^ Tregs, beside the conventional Tregs in the tumor microenvironment.

It was thought that majority of Tregs have memory phenotype, while they were defined by high expression of CD45RO and low expression of CD45RA. Memory Tregs survive longer than naïve Tregs and they have specialized subsets in different tissues (27). CD45RA^+^ Tregs showed increased level of FOXP3 and strong suppressive ability as well as memory Tregs (28). Herein, there was no reduction of CD45RA^+^ lymphocytes after co-culturing with neither cancer nor normal ASCs and the likelihood of increased memory population (CD45RO^+^ Tregs). It seems that the balance between naive and memory Tregs is reversed in some autoimmune diseases, such as multiple sclerosis (MS) showing reduced number and function of CD45RA^+^ Tregs, while expansion of memory Tregs has been reported in these patients (29, 30). Anyway, for achieving more reliable conclusions from our results, it was better to check CD45RO^+^ Tregs or co-culture the cells for a longer period of time.

Helios, is an Ikaros transcription factor family member known as a marker of natural Treg (nTregs). Although recently, several reports have also shown that inducible Tregs (iTregs) express Helios *in vitro* and in vivo (31, 32). Other studies on mixed populations of naïve and memory Helios^+^ or Helios- Tregs showed higher expression of IFN-γ, IL-17 and IL-2 by Helios- Tregs compared to Helios^+^ Tregs (33). In contrast, Himmel et al. (34) revealed that Helios^+^ and Helios^-^ nTregs are not different in their functional properties for suppressing T cell proliferation. In the present study, we investigated the expression of Helios in the population of both CD4^+^CD25^+^FOXP3^+^ and CD4^+^CD25^-^FOXP3^+^ Tregs and the results revealed expansion of this subset in both population after exposing the cells to ASCs, specially to cancer ASCs. Consequently, ASCs not only increase the population of FOXP3^+^ Tregs, but also induce the expression of Helios in these cells. This transcription factor, along with FOXP3, can increase suppressive activity of Tregs and since Helios^+^ cells produce less inflammatory cytokines than Helios- cells (33), the former cells probably show more suppressive activity in the tumor site. The significance of this role of ASCs for inducing Helios is more pronounced when we refer to Yates et al. (35) study. They reported that under the chronic inflammation, Tregs may lose their Helios expression which can result in differentiating to effector T helper cells and consequently suppressing tumor growth.

Tregs mediate their immunosuppressive functions through various mechanisms including cell to cell contact, secretion of IL-35, IL-10 and TGF-β as well as the conversion of adenosine triphosphate (ATP) to adenosine through expression of CD39 and CD73 (36). CD39 and CD73 are two ectonucleotidases that collaborate in the production of extracellular adenosine through ATP hydrolysis. Indeed, CD39 generates adenosine monophosphate (AMP), which is in turn used by the CD73 ectonucleotidase to synthesize adenosine. Consequently, co-expression of CD73 and CD39 on Tregs surface is necessary for the maximum suppressor function (37, 38). In the present study, expressions of CD73 and CD39 were studied when naïve CD4^+^ T cells were co-cultured with ASCs. The results revealed that co-culturing of naïve T cells with ASCs increased CD73^+^CD39^+^, but not CD73^-^ CD39^+^ and CD73^+^CD39^-^ subsets of T cells, which was statistically significant in the presence of cancer-ASCs. Interestingly, CD25- FOXP3^+^CD73^+^CD39^+^ cells were reduced after exposing to both cancer- and normal-ASCs compared to the control group. The results suggest that induced CD25^+^ Tregs in the presence of ASCs, especially cancer-ASCs, may have stronger immunosuppressive effects compared to the CD25- counterparts due to co-expression of CD73 and CD39. This can result in inducing metabolic disruption of the recruited effector T cells to the tumor site. The current results are further confirmed by Saldanha-Araujo et al. (39) who showed that the amount of adenosine and CD73^+^ T lymphocytes augmented significantly after exposing to bone marrow MSCs. Collectively, it can be proposed that adenosine signaling would be important for immunomodulatory properties of ASCs.

According to the results of functional assay obtained from co-cultured naïve T cells, all three cytokines, IL- 10, TGF-β and IL-17 were increased upon co-culturing of naïve T-cells with ASCs. Although cancer-ASCs had more significant effects on developing IL-10- and TGF-β- producing Tregs, normal-ASCs induced IL-17-producing Tregs. Despite most studies indicate secretion of antiinflammatory cytokines by Tregs, it is well demonstrated that specific subgroups of these cells are capable of producing pro-inflammatory cytokines, such as IL-17 with immunosuppressive functions (40).

## Conclusion

Both cancer and normal ASCs create immunomodulatory effects, but it seems that tumor cells educate ASCs for inducing immunosuppressive Tregs. Herein, the obtained results may represent a better understanding of how immune cells and stromal components of tumor site, in particular MSCs, communicate with each other. This can help us predict more successful therapeutic approaches for treatment of breast cancer.
